# Hypodermic Medication in Cholera-Morbus

**Published:** 1879-09-20

**Authors:** John C. Pearson


### *HYPODERMIC MEDICATION IN CHOLERA-MORBUS.*

BY JOHN C. PEARSON, M.D.

It is often said and written that the result of the treatment of isolated cases is frequently of little or no value in deciding upon the efficiency or non-efficiency of any particular line of therapeutic measures. But, on the contrary, when any plan of medication always, or at least very nearly always, is followed by certain definite, favorable and successful effects, we are fully warranted in the conclusion that such effects are the result of the application and use of the plan; that the certain relation of cause and effect is fully established. Most especially may this very justly be claimed when any special plan of treatment has in any special pathological condition been, through long years of trials, uniformly successful.

The writer hereof is led to make these reflections when he takes a long retrospect, of some years, regarding the morbid condition whereto we give the name of cholera-morbus, and his treatment of the same.

Some years ago, during first period of a summer. when Sirius, the dog star, was in the ascendant, and on one excessively hot afternoon, I was sent for to see a German, and a most esteemed and valued friend, who the messenger said was sick as "the tyful, and he go so dead," imitating, as he imparted this information, the actions of a dying man as near as he could, by closing his eyes, throwing back his head and spreading out his hands with a spasmodic jerk!

Upon my arrival at the house of my patient, and a hasty examination, I would at once, and without the least hesitation, have diagnosed the case one of genuine Asiatic cholera had any case of that dread disease been prevailing in the neighborhood at that time; for the patient before me had every characteristic symptom of the same to a high degree—at least, such appeared to be the case upon a first and rapid examination; and it was not until I had made a more thorough inspection of the symptoms that I diagnosed that I had an extremely severe case of cholera-morbus to combat. He was most severely cramped in the abdomen, legs, arms and feet; vomited, almost incessantly, matter looking like soap suds; had frequent and copious evacuations of rice water appearance from the bowels; hands and arms cold to the elbows, and feet and legs cold to the knees; almost pulseless at the wrist; great drops of cold, clammy sweat stood upon his face, and he was suffering excruciating agony, as his complaints and outcries, and extreme restlessness and anxious countenance most clearly indicated.

At that time the little hypodermic instrument had not come to be used so much and frequently as it has since the period whereof I write, and I had never read or heard of it being used in the same; but it occurred to my mind that it *might* give relief; that I would try it; it wouldn't take long to try it, and if it failed, I could resort to other measures. Acting upon this self-made suggestion, I promptly and without delay, injected into the subcutaneous tissue of the arm a half grain of morphia sulph. and the fortieth of a grain of sulph. of atropia, and sat down to wait for a few minutes for the result. I had not long to wait, however, for in five minutes' time the cramps, the vomiting, the purging, the jactitation had all begun to lessen, and in twenty miuutes more *every one and all of the symptoms had ceased entirely.*

Never before had I been so greatly surprised as I was at the effect I had obtained, and for some time expected a return of the symptoms, having no confidence in the permanency of the good I had accomplished; but there was never a return of any of them, and I absolutely gave my patient no other medicine than that aforementioned, excepting a little for a slight consecutive fever of twenty-four hours' duration.

The results obtained in this case were so very much better than I had dared to hope for, they so quickly followed the administration of the remedy that, for some time subsequently, I was disposed to regard the sudden cessation of all the symptoms as a possible coincidence, but yet felt determined to repeat the treatment, the result of which had so greatly surprised me, the next time a similar case should come under my care. And not many days elapsed before an opportunity was afforded me of further trying the same treatment in a similar and nearly as severe a case. The effects that followed were fully as prompt, decided and lasting; and during the remarkably hot weather of that summer and autumn I had frequent opportunities of further testing the efficiency of the hypodermic treatment of cholera-morbus, and it never disappointed me, though occasionally I would have to repeat the dose before I would obtain entire relief for all of the symptoms. And since that time, ten years ago, I have always similarly treated cholera-morbus, and always successfully and permanently so, and generally without the occurrence of consecutive fever.

And for some years past, when I have been summoned to a case of vomiting and purging, I go feeling assured that I will be able to give relief in a short while, with but little trouble and delay, and without bringing into requisition any of the old fashioned and old fogyish therapeutics of past years, such as "broken doses" of calomel, cayenne pepper, rubefacients of turpentine and ammonia, hot bottles, hot bricks, *et id omne genus*, a plan that necessitated twenty-four hours' time, resulted in the patient getting all the skin rubbed off his legs and arms, and sometimes left him with an infernally sore, salivated mouth and swelled tongue, wherewith to curse, in thickened accents, that "d——d doctor" for salivating him so badly!

I have heretofore reported the treatment of one case of cholera-morbus, upon the plan herein mentioned, in the Reporter, but do not recollect of having ever seen any accouuts of similar treatment for that disease by other physicians, though I suppose that others may have adopted and practiced it. I have very often pursued the same plan for the relief of long-continued and obstinate vomiting, the result of different causes, and successfully in most of the cases, especially those cases denominated "bilious vomiting."

[We have for years used morphia hypodermically in cholera-morbus, but never more than ¼ gr. at a time, which we have found sufficient, while it is certainly a safer quantity.—ED.]

